# Miniopen Transforaminal Lumbar Interbody Fusion with Unilateral Fixation: A Comparison between Ipsilateral and Contralateral Reherniation

**DOI:** 10.1155/2016/7261027

**Published:** 2016-11-03

**Authors:** Zheng Li, Fubing Liu, Shuhao Liu, Zixian Chen, Chun Jiang, Zhenzhou Feng, Xiaoxing Jiang

**Affiliations:** Department of Orthopaedics, Zhongshan Hospital of Fudan University, 180 Fenglin Road, Shanghai, China

## Abstract

The aim of this study was to evaluate the risk factors between ipsilateral and contralateral reherniation and to compare the effectiveness of miniopen transforaminal lumbar interbody fusion (TLIF) with unilateral fixation for each group. From November 2007 to December 2014, clinical and radiographic data of each group (ipsilateral or contralateral reherniation) were collected and compared. Functional assessment (Visual Analog Scale (VAS) score and Japanese Orthopaedic Association (JOA)) and radiographic evaluation (fusion status, disc height, lumbar lordosis (LL), and functional spine unit (FSU) angle) were applied to compare surgical effect for each group preoperatively and at final followup. MacNab questionnaire was applied to further evaluate the satisfactory rate after the discectomy and fusion. No difference except pain-free interval was found between ipsilateral and contralateral groups. There was a significant difference in operative time between two groups. No differences were found in clinical and radiographic data for assessment of surgical effect between two groups. The satisfactory rate was decreasing in both groups with time passing after discectomy. Difference in pain-free interval may be a distinction for ipsilateral and contralateral reherniation. Miniopen TLIF with unilateral pedicle screw fixation can be a recommendable way for single level reherniation regardless of ipsilateral or contralateral reherniation.

## 1. Introduction

Recurrent lumbar disc herniation (rLDH) refers to disc herniation occurring at the ipsilateral or contralateral side of previous operation level and causes clinical symptoms after more than six months of “painless” period from primary surgery [1–3]. Many risk factors have been reported to be associated with rLDH, including age, gender, traumatic history, and disc degeneration [1, 4]; however, most risk factors are focused on ipsilateral reherniation, and only a few articles pay attention to the contralateral reherniation. Evidences show that different pathogenic mechanism may exist in those two kinds of reherniation [5, 6].

Recurrent lumbar herniation has become a common reason for revision surgery, and the optimal surgical treatment for rLDH is still controversial. Some authors propose that repeat discectomy is the treatment of choice, which could achieve satisfactory clinical outcome comparable to the primary procedure, and some spine surgeons believe that fusion is a reasonable choice as repeated discectomy requires more removal of disc materials, which would potentially affect the segmental stability; besides, the presence of scar tissue may increase the risk of nerve injury or dural tear [5, 7, 8]. Several articles have reported that TLIF with bilateral fixation is a recommendable choice for rLDH with satisfied surgical effect [8, 9]. Sonmez and Xue analyzed patients who underwent unilateral percutaneous instrumentation plus TLIF for rLDH and compared them with bilaterally instrumented group. Both groups had a significant decrease in VAS and JOA scores after surgery, while unilateral instrumented group had some advantages in operation time, blood loss, and economic cost [10, 11]. Up to now, no articles have been concerned with the difference which may exist between ipsilateral and contralateral reherniation after fusion. The aim of this study is to evaluate the risk factors between ipsilateral and contralateral reherniation and to compare the effectiveness of miniopen transforaminal lumbar interbody fusion (TLIF) with unilateral fixation for ipsilateral reherniation with those for contralateral ones.

## 2. Materials and Methods

### 2.1. Patient Group

**Figure 1:**
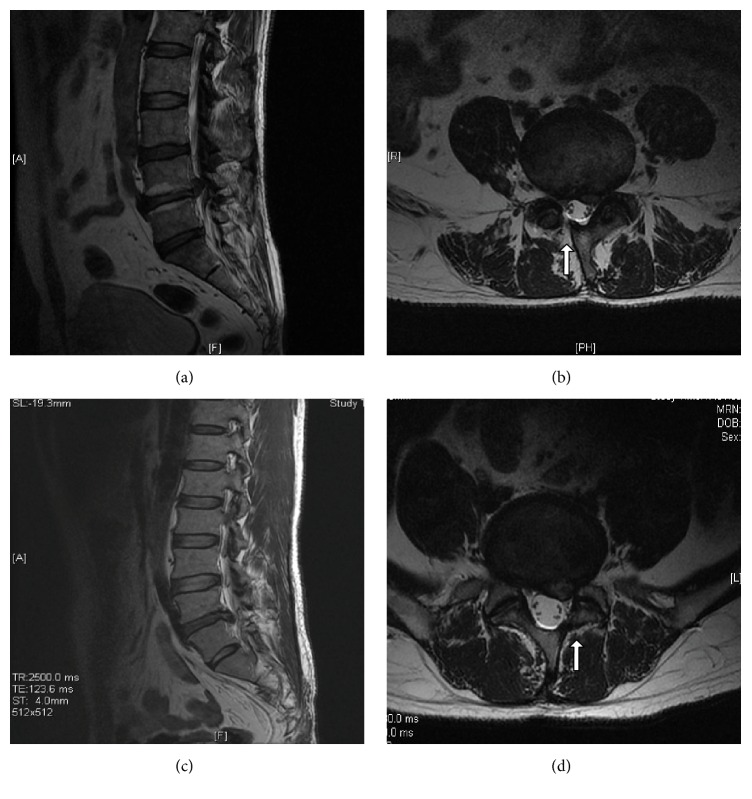
T2 sagittal (a) and axial (b) MRI show contralateral reherniation; T2 sagittal (c) and axial (d) MRI show ipsilateral reherniation. The arrow reflecting the laminectomy defect due to previous surgery.

From November 2007 to December 2014, 38 patients who were treated with unilateral pedicle screw instrumented TLIF were included in this study; among them, 31 patients with ipsilateral reherniation were set as group I and 7 patients with contralateral reherniation were set as group II. The inclusion criteria were (1) recurrent disc herniation nonresponse to conservative treatment of more than 3 months; (2) over 6 months of pain-free period after primary discectomy; (3) ipsilateral or contralateral disc herniation observed on imaging at the same level as the primary discectomy. Patients with pathological vertebral fracture, severe osteoporosis of the spine, active infection, or spinal metastasis were excluded. All patients developed back/leg pain, leg numbness, or intermittent claudication after an initial pain-free interval, which was averaged 63.3 months (range 6–228 months) following discectomy. All cases were single level recurrent herniation with imaging confirmed (Figure 1).

### 2.2. Risk Factors Evaluation

**Figure 2:**
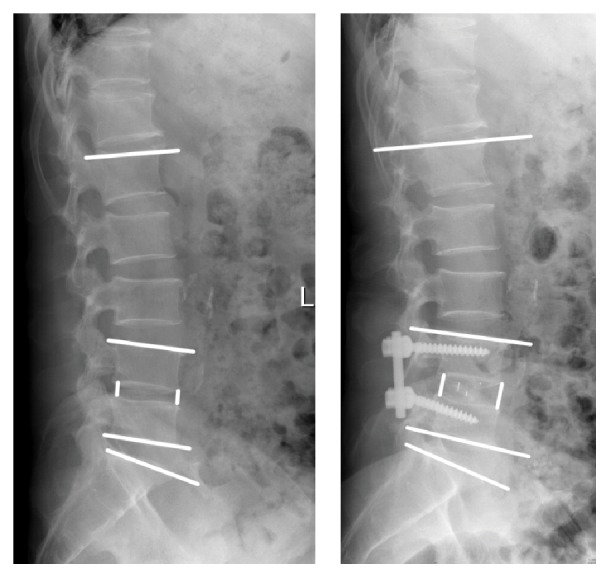
Preoperative and 3-day postoperative anterior-posterior X-ray images showing unilateral pedicle screw instrumented TLIF on L4-5 level. The angle of lumbar lordosis was measured between the superior endplate of L1 and S1. The functional spine unit was measured between the superior endplate and inferior endplate of fusion segment. The mean disc height was defined as the arithmetic mean between anterior and posterior disc height.

The patients were divided into ipsilateral or contralateral group based on the orientation of the reherniation. Demographic and clinical data including age, gender, pain-free interval, LDH types, and traumatic history were compared between two groups. Radiographic factors including disc height (DH), lumbar lordosis (LL), and functional spine unit (FSU) angle were compared; two experienced spine surgeons who were blind to the clinical data took the measurement of radiographic value. DH, LL, and FSU angle were measured as the figure showed (Figure 2).

### 2.3. Surgical Procedure

All operations were conducted by the same surgeon in a single center. After successful general anesthesia, the patient was placed in a prone position, and the surgical level was confirmed with the help of a C-arm machine. A paramedian longitudinal incision about 4 cm long was made on the reherniation side. Paraspinal muscle was split and retracted to expose the articular process, transverse process, and lamina. According to the surface location and anatomic marker, two pedicle screws were placed, and then the inferior and superior articular processes, part of lamina, and ligamentum flavum were removed, to decompress the nerve root, A complete discectomy and end-plate preparation were performed; thereafter, a suitable cage filled with autologous bone, which came from the resected bones, was placed obliquely across the disc space, and then connecting rod was installed, followed by the fluoroscopy confirmation. After washing the wound with saline, a drainage tube was placed, and the incision was sutured by layers.

### 2.4. Surgical Outcome Evaluation

Perioperative parameters of both groups including incision length, intraoperative blood loss, drainage volume, operative time, hospital stay, and postoperative complications were obtained from hospital records and compared. Visual Analog Scale (VAS) score and Japanese Orthopaedic Association (JOA) were applied to assess the pain and functional outcome for each group preoperatively and at final followup. The patients were examined with X-ray films at 2, 6, and 12 months and annually thereafter after surgery. Three-dimensional CT (3DCT) scan was performed at 6 months and yearly to assess the fusion status accurately. Solid fusion was defined as bone bridging the disk space without lucency according to the 3DCT with sagittal and coronal reconstruction [12]. MacNab questionnaire was applied to further evaluate the satisfactory rate after the discectomy and fusion for each group.

### 2.5. Data Analysis

Statistical analysis was performed with SPSS 20.0 (SPSS, Inc., Chicago, IL, USA). The data of ipsilateral and contralateral groups were compared by two independent sample* t*-test and Fisher's exact test. A *P* value < 0.05 was considered to be significant.

## 3. Results

The 38 patients were followed up for a mean duration of 52.2 months, ranging from 12 to 93 months. Risk factors evaluation was shown in Table 1; there was no statistical significance for the two groups in age, gender, and traumatic history. Pain-free interval had significant difference between two groups and contralateral group has longer time of pain-free interval compared to that of ipsilateral group. Protruded and extruded type were more common in ipsilateral reherniation while extruded type except one protruded patient makes up the majority of contralateral reherniation group before the first surgery. Both groups have no sequestered patient. After the discectomy, the ratio of those types has no significant statistic difference (Table 2).

Perioperative parameters including incision length, blood loss, drainage volume, operative time, and hospital time were shown in Table 3. There was no statistical significant difference existing between the ipsilateral and contralateral group regarding the perioperative parameters except the operative time. One patient in ipsilateral group had superficial wound infection after surgery. The situation was under control after several dressing changes. No other complications such as dural tear were observed among these patients during the perioperative period. For radiographic data, DH, LL, and FSU angle and fused segment have no statistical difference for two groups before and after discectomy and after fusion with unilateral fixation TLIF (Table 4). Compared with preoperative values, the lumbar JOA scores of last followup were obviously improved. The postoperative VAS score was obviously lower than that of preoperative. Both groups showed no statistical significance in preoperative and postoperative score and fusion rate (Table 5). The satisfactory rates were decreasing in both groups with time passing. After 6 months from the first surgery, the satisfactory rate was 93.5% (excellent and good according to the Macnab criteria) in ipsilateral group, while in contralateral group the rate was 100% and the rate decreased at 2 years after the discectomy (58.1% in ipsilateral group and 71.4% in contralateral group) and was lower at 4 years after surgery (41.9% in ipsilateral group and 57.1% in contralateral group) (Table 6).

## 4. Discussion

Recurrent lumbar herniation has become a common reason for revision surgery. The incidence of rLDH ranges from 5% to 11% and increases over time [13]. Many risk factors have been reported to be associated with rLDH. Suk et al. reported that young age, male gender, smoking, and traumatic history may be the risk factors for recurrent herniation after conventional open discectomy [1]. In another report, Choi et al. conducted a study which showed that long pain-free interval and mild disc degeneration could differentiate the development of contralateral reherniation from that of ipsilateral reherniation [5]. In our study, pain-free period of contralateral group was significantly longer than that of ipsilateral group as Choi et al. reported. Besides, a longer pain-free time may indicate a high satisfactory rate to some extent. Contralateral reherniation may have a different pathology mechanism from that of ipsilateral. The extruded type was more common in contralateral group, for this type of herniation requires more removal of disc materials which may reduce the rate of rLDH in a short time; however, it may also potentially affect the segmental stability and accelerate the disc degeneration. As a result, rLDH will be induced, and it may play more important role in contralateral reherniation.

Surgical treatment for rLDH has been controversial and can be broadly categorized as revision discectomy alone or revision discectomy with fusion; however, some authors argued that repeat discectomy would weaken the stability of the involved spine and increased the risk of rLDH. Österman et al. in a large retrospective study revealed that patients undergoing multiple revisions after lumbar discectomy got markedly reduced risk for subsequent operations if the first procedure was a spinal fusion [14]. Therefore, it appears to be a reasonable choice for fusing the index level in cases of rLDH. There are many fusing choices including PLF, PLIF, ALIF, and TLIF, the latter of which has been a well-accepted procedure [15]. The use of unilateral pedicle screw fixation with TLIF for rLDH was also reported in some study and the clinical effect was satisfied [10, 11]. Our study demonstrated that JOA and VAS score improved significantly after surgery. Postoperative radiographic result showed a good fusion rate which indicated the effect of this surgical technical. Besides, a miniopen paramedian approach about 4 cm long was applied, which can provide a greater surgical field, and miniopen TLIF required shorter time to learn compared to minimally invasive TLIF [16].

Perioperative parameters data showed no difference between ipsilateral and contralateral group except the operative time, even though the paramedian TLIF can provide a facilitated pathway through the unscarred tissue. However, more attention is still needed to avoid dural rupture or root injury, and this may illustrate the reason for more time in ipsilateral group. Preoperative and postoperative functional evaluation including JOA and VAS had no statistical difference between two groups; the fusion rate was comparable to that of other studies [11, 17]. Satisfactory rate after fusion was quite good at last followup. Taken together, miniopen TLIF with unilateral pedicle screw fixation can be a recommendable way for single level rLDH regardless of ipsilateral or contralateral reherniation.

There are some limitations in this study. Firstly, it is a retrospective case-control study, which inevitably has selection and recall bias, despite the fact that we collected and analyzed the data meticulously. Secondly, the number of patients included in this study is relatively small and the followup time is relatively short in some patients. Thus, a randomized controlled study with enough samples is needed to further confirm the safety and effectiveness of this surgical technology.

## 5. Conclusion

Difference in pain-free interval may be a distinction for ipsilateral and contralateral reherniation. Unilateral pedicle screw instrumented TLIF via a miniopen paramedian incision can provide a safe, effective, and less invasive way for the treatment of rLDH regardless of ipsilateral or contralateral reherniation.

## Figures and Tables

**Table 1:** Risk factors data analysis.

Parameters	Group		*P*
	Ipsilateral (31)	Contralateral (7)	
Age (years)	51.9 ± 11.4	46.3 ± 11.1	0.258
Gender (male/female)	19 : 12	3 : 4	0.425
Pain-free interval (months)	54.3 ± 51.2	102.9 ± 79.0	0.048
Fused segment			
L4-L5	20	2	0.108
L5-S1	11	5	

Data presented as mean ± SD. *P* < 0.05 was considered to be significant.

**Table 2:** Types of lumbar disc herniation distribution of two groups before and after discectomy.

Parameters		Groups		
	LDH types	Ipsilateral (31)	Contralateral (7)	*P*
Before discectomy	Protruded-type	18	1	
	Extruded-type	13	6	0.09
	Sequestered-type	0	0	

After discectomy	Protruded-type	14	3	
	Extruded-type	17	4	1.00
	Sequestered-type	0	0	

Data presented as mean ± SD. *P* < 0.05 was considered to be significant.

**Table 3:** Perioperative parameters in patients undergoing unilateral fixation TLIF for the treatment of recurrent herniation.

Variable	Ipsilateral (31)	Contralateral (7)	*P*
Incision length (cm)	4.0 ± 0.3	4.1 ± 0.2	0.408
Intraoperative blood loss (mL)	119.5 ± 78.4	75.7 ± 36.5	0.161
Drainage volume (mL)	118.6 ± 82.6	174.3 ± 111.7	0.139
Operative time (minutes)	86.8 ± 18.9	70.0 ± 17.3	0.038
Hospital time (days)	9.7 ± 3.1	9.3 ± 1.7	0.713

Data presented as mean ± SD. *P* < 0.05 was considered to be significant.

**Table 4:** Radiographic evaluation before and after discectomy and after fusion with unilateral fixation TLIF for the treatment of recurrent herniation.

Period	Parameters	Groups		*P*
		Ipsilateral (31)	Contralateral (7)	
Before discectomy	Disc height	11.2 ± 1.0	10.6 ± 1.2	0.211
	Functional spine unit	16.8 ± 4.9	13.6 ± 2.9	0.113
	Lumbar lordosis	34.2 ± 8.1	36.6 ± 4.9	0.468

After discectomy	Disc height	11.1 ± 2.1	11.8 ± 1.5	0.404
	Functional spine unit	16.5 ± 4.8	15.0 ± 5.9	0.472
	Lumbar lordosis	34.8 ± 9.2	38.5 ± 5.9	0.322

After fusion	Disc height	12.4 ± 1.8	13.5 ± 1.9	0.170
	Functional spine unit	17.0 ± 5.8	12.5 ± 3.4	0.054
	Lumbar lordosis	35.6 ± 10.5	33.3 ± 7.5	0.590

Data presented as mean ± SD. *P* < 0.05 was considered to be significant.

**Table 5:** Clinical evaluation before and after unilateral fixation TLIF for the treatment of recurrent herniation.

Variable	Ipsilateral (31)	Contralateral (7)	*P*
Preoperative			
Back pain VAS score	6.3 ± 1.5	6.8 ± 1.4	0.403
Leg pain VAS score	7.7 ± 0.7	8.0 ± 0.4	0.280
JOA score	9.0 ± 1.7	8.4 ± 1.4	0.367
Last followup			
Back pain VAS score	1.0 ± 0.7	1.1 ± 0.7	0.709
Leg pain VAS score	1.2 ± 0.9	1.4 ± 0.5	0.473
JOA score	26.2 ± 1.3	26.0 ± 0.6	0.614
Fusion rate	90.3%	85.7%	1.000

Data presented as mean ± SD, *P* <0.05 was considered to be significant.

**Table 6:** Satisfactory rate for two groups after the discectomy and fusion.

Satisfactory rate	Time	Groups	
		Ipsilateral (31)	Contralateral (7)
After the discectomy	6 months	93.5%	100%
	2 years	58.1%	71.4%
	4 years	41.9%	57.1%

After the fusion	6 months	100%	100%
	Last followup	96.8%	100%
